# Maternal antibiotic administration during gestation can affect the memory and brain structure in mouse offspring

**DOI:** 10.3389/fncel.2023.1176676

**Published:** 2023-05-10

**Authors:** Dmytro Shepilov, Iryna Osadchenko, Tetiana Kovalenko, Chiaki Yamada, Anastasiia Chereshynska, Kateryna Smozhanyk, Galyna Ostrovska, Stanislav Groppa, Alexandru Movila, Galyna Skibo

**Affiliations:** ^1^Department of Cytology, Bogomoletz Institute of Physiology, NAS of Ukraine, Kyiv, Ukraine; ^2^Department of Biomedical Sciences and Comprehensive Care, School of Dentistry, Indiana University, Indianapolis, IN, United States; ^3^Indiana Center for Musculoskeletal Health, Indiana University School of Medicine, Indianapolis, IN, United States; ^4^Department of Cytology, Histology, and Reproductive Medicine, Institute of Biology and Medicine, Taras Shevchenko National University of Kyiv, Kyiv, Ukraine; ^5^Department of Neurology, Institute of Emergency Medicine, Chisinau, Moldova; ^6^Department of Neurology, State University of Medicine and Pharmacy “Nicolae Testemiţanu”, Chisinau, Moldova

**Keywords:** offspring mice, antibiotics, memory and learning, hippocampal structure, neurogenesis, myelination

## Abstract

Maternal antibiotics administration (MAA) is among the widely used therapeutic approaches in pregnancy. Although published evidence demonstrates that infants exposed to antibiotics immediately after birth have altered recognition memory responses at one month of age, very little is known about *in utero* effects of antibiotics on the neuronal function and behavior of children after birth. Therefore, this study aimed to evaluate the impact of MAA at different periods of pregnancy on memory decline and brain structural alterations in young mouse offspring after their first month of life. To study the effects of MAA on 4-week-old offspring, pregnant C57BL/6J mouse dams (2–3-month-old; *n* = 4/group) were exposed to a cocktail of amoxicillin (205 mg/kg/day) and azithromycin (51 mg/kg/day) in sterile drinking water (daily/1 week) during either the 2nd or 3rd week of pregnancy and stopped after delivery. A control group of pregnant dams was exposed to sterile drinking water alone during all three weeks of pregnancy. Then, the 4-week-old offspring mice were first evaluated for behavioral changes. Using the Morris water maze assay, we revealed that exposure of pregnant mice to antibiotics at the 2nd and 3rd weeks of pregnancy significantly altered spatial reference memory and learning skills in their offspring compared to those delivered from the control group of dams. In contrast, no significant difference in long-term associative memory was detected between offspring groups using the novel object recognition test. Then, we histologically evaluated brain samples from the same offspring individuals using conventional immunofluorescence and electron microscopy assays. To our knowledge, we observed a reduction in the density of the hippocampal CA1 pyramidal neurons and hypomyelination in the *corpus callosum* in groups of mice *in utero* exposed to antibiotics at the 2nd and 3rd weeks of gestation. In addition, offspring exposed to antibiotics at the 2nd or 3rd week of gestation demonstrated a decreased astrocyte cell surface area and astrocyte territories or depletion of neurogenesis in the dentate gyrus and hippocampal synaptic loss, respectively. Altogether, this study shows that MAA at different times of pregnancy can pathologically alter cognitive behavior and brain development in offspring at an early age after weaning.

## 1. Introduction

Since the COVID-19 pandemic was declared in December 2019, the World Health Organization and international statics, including those from Ukraine, have confirmed that pregnant women are a potentially vulnerable population to secondary bacterial co-infection (Emanoil et al., 2021; Januszek et al., 2022). Therefore, a significant increase in the maternal antibiotics administration (MAA) for therapeutic and prophylactic treatments of various secondary infections in pregnant women infected with COVID-19 (Ramírez-Lozada et al., 2022). However, a recently conducted population-based cohort study demonstrated increased severity of COVID-19 disease after treatment with antibiotics (Llor et al., 2021).

Maternal antibiotics often coincided with the perinatal period to prevent infectious morbidity and mortality (Cardetti et al., 2020; Thinkhamrop et al., 2021) and postpartum infection after post-surgical C-section complications (O’Connor et al., 2021). While the administration of antibiotics is maybe critical to maintaining maternal health during pregnancy, registry-based cohort studies demonstrated that maternal antibiotics exacerbate the risks of developing asthma and early-onset sepsis in their children (Stokholm et al., 2014; Zhou et al., 2020). Furthermore, the MAA can produce long-lasting effects on the neuroimmune responses of offspring in an experimental mouse model of dysbiosis (Madany et al., 2022a).

The normal gut microbiota is thought to be linked to physiological brain development, behavior, and stress response in a healthy lifespan (Heijtz et al., 2011; Dinan and Cryan, 2017). By contrast, dysbiosis of the gut microbiota plays a crucial role in the pathogenesis of several immunological diseases and neuroinflammation in adulthood (Carding et al., 2015; Cao et al., 2021). However, the *in utero* impact of antibiotics on the offspring begins to be explored. Accumulated lines of evidence indicate that MAA in pregnant women affects the gut microbiota and may cause endotoxemia (Madany et al., 2022a; Norooznezhad et al., 2022). Endotoxins are released from dead bacterial cells, which can cross the epithelial barrier ending up in the bloodstream (André et al., 2019). Recently published studies reported that bacterial-derived endotoxins promote neuroinflammation and affect cerebral circulation in pregnant mice (Cipolla et al., 2011; Kurita et al., 2020). Furthermore, endotoxemia mediates perinatal neuroinflammation in a murine model of preterm labor (Estrada et al., 2020). In addition, our recently published study demonstrated that bacterial-derived endotoxins influence hallmark findings in Alzheimer’s disease (Yamada et al., 2020).

Although various published studies addressed the impact of MAA on gut microbiota during pregnancy (Kuperman and Koren, 2016; Madany et al., 2022a,b), it remains unclear whether widely used antibiotics in gynecological practice, *e.g.*, amoxicillin and azithromycin (Andrade et al., 2004; Bookstaver et al., 2015), elevate neuroinflammation and neurodegeneration in offspring after birth. Thus, this study assesses how an antibiotics cocktail containing amoxicillin and azithromycin used during the 2nd or the 3rd week of pregnancy alters offspring outcomes by investigating their behavioral defects and brain structure one month after birth.

Our results indicate that exposure of pregnant females to amoxicillin and azithromycin in drinking water during the 2nd week of pregnancy dramatically elevates intestinal barrier permeability and has a positive trend on the permeability of the blood–brain barrier (BBB) compared to the control group of dams. Furthermore, the density of the hippocampal CA1 pyramidal neurons in groups of offspring exposed to antibiotics at the 2nd and 3rd weeks of gestation was significantly reduced, and myelination in the corpus callosum was altered compared to the control group. In addition, offspring exposed to antibiotics *in utero* at the 2nd or the 3rd week of gestation demonstrated a decreased astrocyte cell surface area and astrocyte territories or depletion of neurogenesis in the dentate gyrus and hippocampal synaptic loss, respectively. Finally, we also confirmed that the administration of amoxicillin and azithromycin cocktail to dams affects spatial reference memory and hippocampus-related spatial learning in offspring.

## 2. Materials and methods

### 2.1. Mice and experimental cohorts

Dams and sires (2–3-month-old; C57BL/6J) mice were purchased from Charles River Laboratory (Czechia) and were exposed to the same sterile drinking water. The breeding protocol was started after one week of acclimatization to a new animal facility. The embryonic (E) day was determined by the presence of a vaginal plug marking E0.5 of pregnancy. Then, pregnant dams were placed individually and randomly divided into three experimental groups (*n* = 4 mice/group). Group I: *control*, no antibiotics in sterile drinking water over the entire period of gestation. Group II (*2-w antibiotics)*, was exposed to an antibiotic pharmaceutic grade cocktail (Basalt, Ukraine) containing a mixture of amoxicillin (205 mg/kg bw/day) and azithromycin (51 mg/kg bw/day) in drinking water starting from the second week of pregnancy (E8-E14); pregnant dams were treated by the antibiotic cocktail in drinking water for 7 days followed by sterile drinking water until birth. Dams from the Group III: (*3-w antibiotics*) received antibiotics cocktail in drinking water from the third week of pregnancy to delivery (E15-P0). Experimental doses of antibiotics were selected according to the average clinical therapeutic doses for adult patients (Smith et al., 2020; Ahmadi et al., 2021) and converted to equivalent ones for adult mice (Nair and Jacob, 2016). Aqueous solutions of antibiotics were changed every other day.

All experimental groups of dams and sires as well as their offspring were kept on a 12-h light-dark cycle at a constant temperature, with free access to food and water. All procedures with experimental animals were carried out in compliance with NIH and Europe community (Directive 2010/63/EU) policies, and ethical approval was obtained from the Biomedical ethics committee at the Bogomoletz Institute of Physiology (protocol #1/22 from August 10, 2022).

**FIGURE 1:**
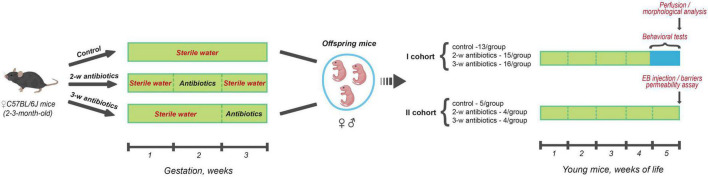
Scheme of the experiment. Pregnant C57BL/6J mice (between 2 and 3-month-old) were divided into three groups (4 females/group) according to the treatment procedure: (I) sterile drinking water over the entire gestation period (*control*); (II) sterile drinking water contained a cocktail of antibiotics (amoxicillin and azithromycin) during the 2nd week (8-14 days) of gestation (*2-w antibiotics*); (III) consumed antibiotics from the 15th day of gestation to delivery (*3-w antibiotics*). In the present study, offspring mice born from those dams were investigated. In doing so, the first cohort of the animals of all three experimental groups performed behavioral tests during the 5th week of life with further morphological analysis of their brains. There also assessed the permeability of the intestinal and blood-brain barriers in mice from the second cohort using Evans Blue extravasation method.

The offspring mice were used to conduct behavioral tests during the 5th week of life followed by morphological analysis of their brains (*n* = 13–16 mice/group). In addition, we determined the BBB and intestinal barrier permeability (n = 4–5 mice/group) as demonstrated in the study design protocol (Figure 1).

### 2.2. Behavioral testing

Since previously published *in utero* pre-clinical observations recommended to at least 4-week-old offspring for behavioral alterations and brain histopathological studies (Zheng et al., 2013; Barnhart et al., 2015; Snow et al., 2015), we conducted our behavioral tests using 4-week-old mice born from experimental Groups I-III.

#### 2.2.1. Novel object recognition test

The novel object recognition (NOR) test is a widely used cognitive task for assessing associative memory (Lueptow, 2017). The assay was conducted in a square plexiglass chamber (40 cm × 40 cm × 40 cm) and comprised three phases: habituation, familiarization, and test. During habituation, mice freely explored an empty room without objects for 5 min. After 24 h, two identical objects—sand-filled glass jars or rectangular wooden blocks—were placed in the device, and mice were allowed to examine them for 10 min (familiarization). Next, the object recognition memory was tested after 24 h. To do that, one of the familiar objects was replaced with a novel objective and explored for 10 min by offspring. To avoid any olfactory cues, the apparatus was cleaned with 70% ethanol between trials. In addition, object types in the familiarization phase and object positions (left/right) in the test phase were counterbalanced between mice. All trials of the familiarization and test phases were analyzed using ANY-maze software, version 7.15 (Stoelting Co., Ireland) using standard protocol. Briefly, the first 5 min of a trial were analyzed; if the mouse did not reach a 20s criterion of object exploration, the next 5 min of the video were also analyzed. Data from mice that failed to reach the standard for the whole 10-min trial were excluded. Preference of the 2nd object during familiarization (*(time with the 2nd object/total time exploring two things) × 100%*) and Preference index (*(time with the novel object/total time exploring novel and familiar objects) × 100%*) were calculated.

#### 2.2.2. Morris water maze

The next day after NOR test, spatial reference memory was evaluated the by Morris water maze (MWM) test using an established protocol with minor modifications (Patil et al., 2009). Mice were trained in a water-filled pool (115 cm in diameter; 21–23°C), and adding dry milk was to make the water opaque. A drape surrounded the pool where spatial cues (geometric figures) were mounted. MWM consisted of three stages: pre-training (day 0), acquisition (days 1–4), and probe test (day 5). During pre-training, animals were habituated to the testing conditions. There were four trials of up to 120 s to find a visible platform (11 cm in diameter, 1 cm above the water) with a 60-min intertrial interval. The start point was fixed (E), while the position of the platform was changed between trials (S-E → S-W → N-W → N-E). After finding the platform, mice were allowed to explore it for the 30 s before returning to home cages. If a mouse did not see the platform for 120 s, it was sat there by the experimenter. The acquisition stage lasted for four consecutive days (4 trials/day, 60-min intertrial interval), during which animals found a hidden platform (0.5 cm below the water). The start point varied between trials (*e.g.*, E → N → W → S), however, the platform’s position was fixed on all days of acquisition (N–W). If a mouse did not reach the platform for the 120 s, the investigator placed it there for the 30 s. On the last day of MWM, a probe test was carried out. The platform was removed from the pool, and animals were allowed to swim for the 60 s. Latency to find the hidden platform, path length, and mean speed during acquisition, as well as time in the target quadrant and heat maps with trajectories of movement in the probe task, were obtained using ANY-maze software, version 7.15 (Stoelting Co., Ireland).

### 2.3. Morphological analysis of the brain

#### 2.3.1. Sample collection

Sample collections from the offspring was performed immediately after the MWM test using an established protocol (Goncharova et al., 2015; Marungruang et al., 2020). Briefly, mice were anesthetized with intramuscular injection of ketamine (100 mg/kg bw; Ketalar, Pfizer AB, Sweden) and xylazine (10 mg/kg bw; Sedazine, Biovet Pulawy, Poland). Then, anesthetized mice were transcardially perfused with a warm (+37^°^C) solution containing 0.1 M PBS (pH 7.4) + 0.3% heparin followed by ice-cold fixative solution containing 4% paraformaldehyde (PFA) + 0.25% glutaraldehyde (GA) (Sigma-Aldrich, USA) in 0.1M PBS. After that, the brains were dissected and split into two hemispheres. Finally, samples for immunohistochemistry (IF) were postfixed overnight in the fixative solution containing 4% PFA at + 4°C. The other brain hemisphere was postfixed in the solution containing 4% PFA + 2.5% GA for light and transmissional electron microscopy.

#### 2.3.2. Immunohistochemistry and confocal microscopy

Altogether, 13–16 samples/treatment group were submitted for immunohistochemistry. The 40-μm-thick coronal sections of the dorsal hippocampus were obtained from the anatomical areas between 1.65 and 2.48 mm posterior to the bregma (Lein et al., 2007) using a vibratome Leica VT1000A (Leica Biosystems, Germany).

To visualize the hippocampal cellular structure, free-floating sections were placed in wells of 24-well plates, rinsed with 0.1M PBS, and treated with a blocking solution containing 1% BSA (Sigma-Aldrich, USA) and 0.3% Triton X-100 (Sigma-Aldrich, USA) for 1h at room temperature. The following primary antibodies were applied for immunostaining: anti-mouse monoclonal antibodies against neuronal nuclei marker (NeuN; 1:750, Abcam, UK), anti-rabbit polyclonal antibodies against the glial fibrillary acidic protein (GFAP, astrocyte marker; 1:1,500, Dako, USA), and anti-rabbit monoclonal antibodies against ionized calcium-binding adaptor molecule 1 (Iba1, microglia/macrophage-specific protein; 1:500, Wako, Japan). Incubation with primary antibodies lasted 16h at +4°C. After rinsing, sections were incubated with secondary antibodies for 1.5 h at room temperature in the dark: donkey anti-mouse Alexa Fluor 594, donkey anti-rabbit Alexa Fluor 647, and donkey anti-rabbit Alexa Fluor 488 (1:1,000, Invitrogen, USA). The sections were then rinsed, placed on histological slides, and mounted with Shandon Immu-Mount medium (Thermo Scientific, USA). Images of hippocampal tissue were taken with an FV1000-BX61WI confocal microscope at 20× (NA – 0.4) and 40× (NA – 0.65) objective magnifications (Olympus Corp., Japan).

To determine the intensity of postnatal neurogenesis, animals were intraperitoneally injected with 5-Bromo-2’-deoxyuridine in saline (BrdU, 50 mg/kg bw; Sigma-Aldrich, USA) two consecutive days before the perfusion (twice a day, at 10:00 and 17:00). 40-μm brain coronal sections (obtained as described above) were pre-treated with a 2N HCl for DNA denaturation during 1.5h at +37°C. After that, a standard immunohistochemical protocol was employed. Goat polyclonal antibodies against doublecortin (DCX, 1:250; Santa Cruz Biotechnology, USA) were used to detect neuroblasts. The proliferatively active cells were identified with rat monoclonal antibodies against BrdU (1:150; Bio-Rad AbD Serotec, UK). Donkey anti-goat Alexa Fluor 488 and goat anti-rat Alexa Fluor 546 secondary antibodies (1:1,000, Invitrogen, USA) were taken to display a marker-specific fluorescence. Confocal images of the dentate gyrus were obtained at 20× (NA – 0.4) objective magnification.

#### 2.3.3. Light and transmission electron microscopy

As described above, collected hemispheres (*n* = 6 samples/condition) were postfixed overnight in a solution containing 4% PFA and 2.5% GA at +4°C. Next day, sample were histologically sliced into 300-μm-thick coronal sections using the vibratome Leica VT1000A (Leica Biosystems, Germany) on the levels of the hippocampus (bregma between −1.66 and −2.48 mm) and corpus callosum (bregma between 1.05 and −0.96 mm) (Lein et al., 2007). Samples were placed in 1% OsO_4_ for 1 h, then dehydrated in an ascending series of ethanol, followed by dry acetone, and embedded in EPON resin (Sigma-Aldrich, Switzerland) according to a standard protocol (Weakley, 1972).

To quantify the hippocampal pyramidal neurons, semi-thin (1 μm) sections were prepared with an LKB 8800 ultramicrotome (LKB, Sweden), stained with methylene blue, embedded in a Pertex mounting medium (HistoLab Products AB, Sweden), and visualized using a Carl Zeiss Axiolab Re light microscope (Carl Zeiss AG, Germany) (objective magnification – 20×, NA – 0.40). For ultrastructural analysis, ultra-thin sections (60–70 nm) from the middle part of the CA1 *stratum radiatum* or corpus callosum were contrasted with uranyl acetate and lead citrate and examined using a JEM-100CX transmission electron microscope (Jeol, Japan) at magnifications of ×10,000 (hippocampal neuropil) or ×4,800 and ×6,400 (myelinated axons).

#### 2.3.4. Quantifications and measurements

The brain morphology was performed using the ImageJ software, version 1.53k (https://imagej.nih.gov/ij/index.html; NIH, USA), and MyelTracer software (Kaiser et al., 2021) by a blind investigator. Quantitative parameters of the hippocampal neurons and glia – number density of neurons per mm^2^ of the *stratum pyramidale*, number densities of GFAP^+^ astrocytes and Iba1^+^ microglia per mm^2^ of *stratum pyramidale* and *stratum radiatum*, glial cell surface area, astrocyte territory (area of brain tissue covered by individual astrocyte), the number of astrocytic and microglial base processes – were manually estimated in 3-5 sections/animal. The levels of postnatal neurogenesis in the dentate gyrus were assessed semi-automatically in 3-5 sections/mouse based on the integrated density of DCX^+^ pixels per mm^2^ of the subgranular zone and *stratum granulosum* and BrdU/DCX ratio. For this purpose, images were transformed to 8-bit, and a fixed background threshold was adjusted. Integrated density was calculated by multiplying the mean gray value of DCX^+^ pixels above the background threshold with their total area within the region of interest (ROI). At the same time, BrdU/DCX ratio was obtained by dividing the overall area of BrdU^+^ pixels by the total area of DCX^+^ pixels within the ROI. Structural synaptic plasticity was manually evaluated on electron micrographs of the hippocampus (at least 20/animal) by such parameters: number density of synapses and their different morphological types – simple, perforated, and multiple ones per 100 μm^2^ of the *stratum radiatum*, as well as the postsynaptic density length. Synapses were categorized into morphological groups as previously described (Shepilov et al., 2022), and their representative images are provided in Supplementary Figure 1. To characterize myelination in the *corpus callosum*, the density of myelinated axons was quantified on at least 20 micrographs/animal (magnification x4800). In addition, the diameter of myelinated axons (minimum 100/mouse) and G-ratio (minimum 50/mouse) were measured at magnification x6400. G-ratio, the ratio of the inner and outer radii of a myelinated axon (Mohammadi et al., 2015), is an inversely proportional indicator of the myelin sheath thickness. It was obtained using MyelTracer software as described elsewhere (Kaiser et al., 2021).

### 2.4. Evaluation of the intestinal barrier and blood-brain barrier (BBB) permeability

To study the physiological barrier permeability, a modified Evans Blue (EB) extravasation method was applied as described elsewhere (Uyama et al., 1988; Alves da Silva et al., 2011). Briefly, offspring mice were intravenously injected with 2% EB solution (4 mg/kg bw) in PBS. After 2 h, mice were anesthetized and perfused with an ice-cold 0.1M PBS. Then, brains and ileum tissue were isolated and homogenized in 0.5 ml of 0.1 M PBS, followed by adding 0.5 ml of 50% trichloroacetic acid. Specimens were vortexed for 2 min and left for 24 h at + 4°C to precipitate proteins. Next day, samples were centrifuged at 10,000 g in an Eppendorf 5415R refrigerated centrifuge (Eppendorf AG, Germany) for 20 min at +4°C. The EB absorbance was measured in supernatants at 610 nm and compared against a calibration curve (serial dilutions of the stock dye solution, concentration range is 0.1-50 μg/ml) using an LLG-uniSPEC2 spectrophotometer. Negative control values (homogenates of tissues from intact animals) normalized per unit mass were subtracted from sample values to obtain the final levels of EB leakage in the brain and intestine. The results were expressed as ng of EB/mg of tissue.

### 2.5. Statistical analysis

Statistical analysis was performed in the GraphPad Prism, version 8.0.2 (GraphPad Software, Inc., USA). The normality distribution of experimental data was evaluated using the Shapiro–Wilk normality test, and the equality of variances was determined with Levene’s test. Data conformed to the Gaussian distribution were described as the Mean ± SEM and processed using the one-way ANOVA followed by Dunnett’s *post hoc* test. Meanwhile, data with non-Gaussian distribution were presented as Median and interquartile range (IQR, 25th–75th percentile). Comparative statistics, in that case, was performed with the Kruskal–Wallis test, followed by Dunn’s *post hoc* test. Relationship between variables was assessed using the Pearson correlation for data with normal distribution and the Spearman correlation for non-parametric variables. R value of 0–0.29, 0.3–0.49, and >0.5 indicates weak, moderate, and strong correlation, respectively. Differences between groups were considered statistically significant when *p* < 0.05.

## 3. Results

### 3.1. Long-term associative memory

First, we investigated associative recognition memory using the NOR test. During the familiarization phase, animals of all groups spent equal time with each identical object, as indicated by the exploratory preference for the 2nd object ranged between 49.2 and 52.9% (Supplementary Figure 2A). Nevertheless, a 24-h delay appeared to be a long period for maintaining a memory trace in young mice. The preference index, which characterizes a relative time exploring the novel object, stood at 49.4 ± 6.1, 46.4 ± 3.9, and 49.7 ± 4.7% for the control, 2-w antibiotics, and 3-w antibiotics groups in that order (Supplementary Figure 2B).

### 3.2. Spatial memory and learning

**FIGURE 2:**
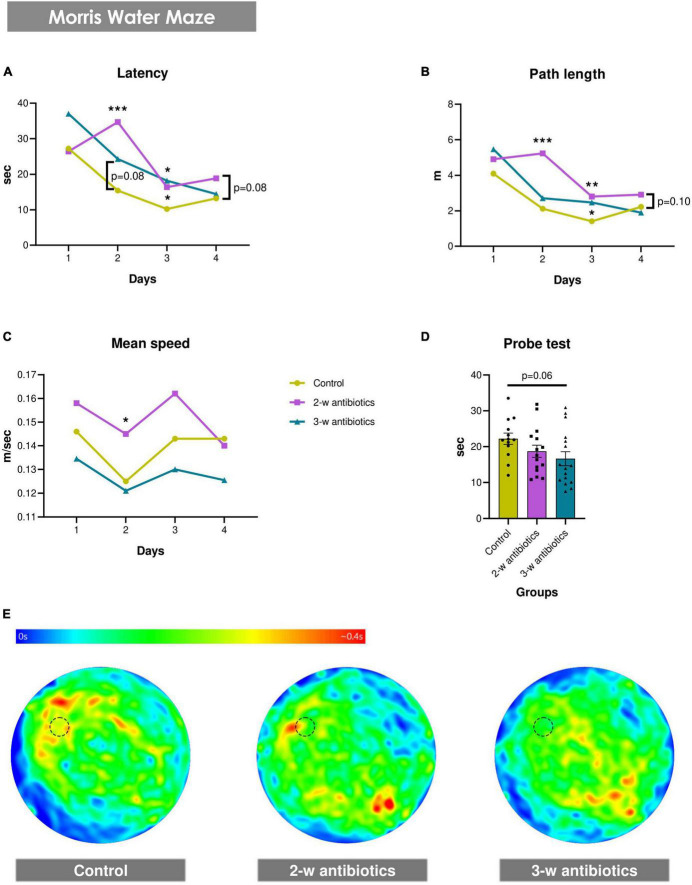
Spatial memory and learning in young mice born to antibiotic-treated C57BL/6J females. **(A–E)** Morris water maze test: **(A)** latency to find a hidden platform, **(B)** path length, and **(C)** mean speed during the acquisition task, as well as **(D)** time in the target quadrant of the water pool and **(E)** heat maps with animals’ trajectories in the probe test. Dotted line circle on the heat map corresponds with the position of the hidden platform. Data on panels (A–C) are expressed as the Median with IQR, while data on graph 2D are expressed as the Mean ± SEM and presented in the form of dot plots (n = 13-16/group). Comparisons among groups were performed with either the one-way ANOVA followed by Dunnett’s *post hoc* test (data with Gaussian distribution) or the Kruskal-Wallis test followed by Dunn’s *post hoc* test (data with non-parametric distribution). *p < 0.05, **p < 0.01, and ***p < 0.001.

Next, we evaluated whether offspring exposed to antibiotics *in utero* demonstrated pathological changes in their spatial memory and learning alterations. Using the MWM test, we observed that the MAA significantly affected spatial reference memory and the hippocampus-related spatial learning in offspring mice (Figure 2). It was revealed that latency to find the hidden platform increased by 124% (*p* < 0.001) and 59% (*p* < 0.05) in the 2-w antibiotics group and by 57% (*p* = 0.08) and 77% (*p* < 0.05) in the 3-w antibiotics group on the 2nd and 3rd days of acquisition, respectively, compared with the control group. In addition, a tendency (*p* = 0.08) to extend the time necessary to reach the platform on the 4th day of testing was traced in 2-w antibiotics animals (Figure 2A). Similar to these results, 2-w antibiotics mice traveled 2.47 (*p* < 0.001) and 1.98 (*p* < 0.01) times longer distances on the 2nd and 3rd days of acquisition than their control peers. In contrast, 3-w antibiotics animals had 1.75 times greater (*p* < 0.05) path length on the 3rd day of the MWM test compared to controls (Figure 2B). Interestingly, 2-w antibiotics mice developed the highest speed in MWM, displaying a mean speed value during the 2nd day of testing 16% more (*p* < 0.05) than the same indicator in control animals (Figure 2C). It should be also noted that offspring born form the 3-w antibiotics group dams memorized a location of the platform worse than others, spending 25% less time (*p* = 0.06) in the target quadrant during the probe test versus control animals (Figures 2D, E).

### 3.3. Functional integrity of physiological barriers

Because behavioral changes mediated by antibiotics often correlate with dysfunction of various blood-tissue barriers permeability (Kwon H. et al., 2020; Ray et al., 2021), we tested next the integrity of the intestinal barrier and blood-brain barrier (BBB) using the Evans blue (EB) leakage assay. It was detected that offspring born from the 2-w antibiotics group of dams had 2.45 times more EB concentration in the ileum tissue than those of the control group (*p* < 0.05; Supplementary Figure 3A). Furthermore, we also observed an elevated tendency for BBB disruption in offspring from the 2nd week of antibiotics exposure group (*p* = 0.07; Supplementary Figure 3B). In contrast, no or little effect on BBB leakage was observed in offspring collected from the group of dams exposed to antibiotics at the 3rd week of pregnancy (Supplementary Figure 3B).

### 3.4. Neuropathological changes in the hippocampal CA1 area

#### 3.4.1. Hippocampal CA1 pyramidal neurons

**FIGURE 3:**
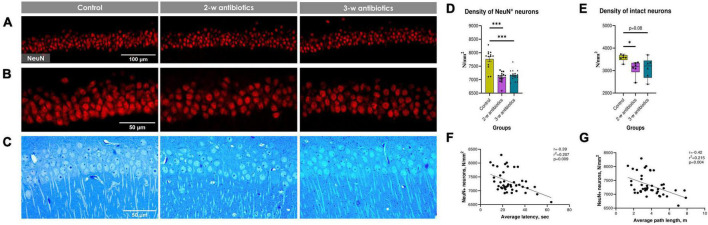
Hippocampal CA1 pyramidal neurons of the experimental mice. **(A,B)** Immunohistochemical images of NeuN^+^ neurons in the CA1 *stratum pyramidale* at **(A)** 20× and **(B)** 40× objective magnifications. **(C)** Methylene blue-stained semi-thin sections of the *stratum pyramidale* used for light microscopic analysis (40x objective magnification). **(D)** The number of NeuN^+^ cells per mm^2^ of the pyramidal layer. **(E)** The number of intact (normochromic) neurons per mm^2^ of the pyramidal layer on semi-thin sections. **(F,G)** Correlational analysis between the density of NeuN^+^ cells and **(F)** average latency, **(G)** average path length during the acquisition task in MWM. Data on panel (D) are expressed as the Mean ± SEM and presented in the form of dot plots, whereas data on panel (E) are expressed as the Median with IQR and visualized using box and whisker plots (*n* = 6/group). Comparisons among groups were performed with either the one-way ANOVA followed by Dunnett’s *post-hoc* test (data with Gaussian distribution) or the Kruskal–Wallis test followed by Dunn’s *post hoc*-test (data with non-parametric distribution). *p < 0.05 and ***p < 0.001.

Since dysbiosis-mediated inflammation in early life leads to increased dysfunction of CA1 pyramidal neurons in children and adults (Hagberg et al., 2012; Gomez et al., 2019), we tested next the NeuN^+^ neuronal density in the CA1 *stratum pyramidale* using confocal microscopy assay (Figures 3A, B). To our knowledge, the density of NeuN^+^ pyramidal neurons in offspring from the 2-w antibiotics and 3-w antibiotics groups was diminished (*p* < 0.001) compared to mice born from the control group of dams (Figure 3D).

Next, we evaluated the density of morphologically intact neurons, *i.e.*, neuronal cells with light blue nuclei, visible 1–3 nucleoli, thin normochromic cytoplasm, and clearly defined apical dendrites, using the light microscopy assay. To our knowledge, the density of morphologically intact neurons was also diminished in 2-w antibiotics (*p* < 0.05) and 3-w antibiotics (*p* = 0.08) compared to the control group (Figures 3C, E).

Finally, we aimed to find a possible correlation between MWM behavioral alterations and the number of NeuN^+^ neurons in the hippocampus. Surprisingly, our data demonstrate a significant negative correlation between the diminished number of NeuN^+^ neurons and average latency (*r* = −0.39; Figure 3F), and path length (r = −0.42; Figure 3G).

#### 3.4.2. Morphological changes in astroglia and microglia in the hippocampus

**FIGURE 4:**
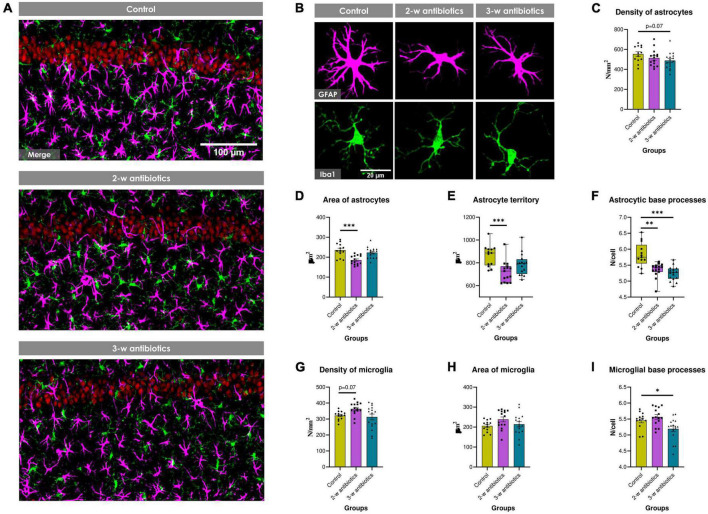
Glial cells in the hippocampus of young offspring animals. **(A)** Immunofluorescent-labeled images of the CA1 *stratum pyramidale* and *stratum radiatum* at 20× objective magnification: red—NeuN^+^ neurons, green—Iba1^+^ microglia, magenta—GFAP^+^ astrocytes. **(B)** Images of individual glial cells at higher objective magnification (40×) displaying fine morphology of cellular somas and processes: magenta—GFAP^+^ astrocytes, green—Iba1^+^ microglia. **(C–I)** Morphometric indicators of hippocampal glia: the number density of **(C)** astrocytes and **(G)** microglial cells per mm^2^ of brain tissue, the cell surface area of **(D)** astrocytes and **(H)** microglia, **(E)** astrocyte territory, as well as the mean number of **(F)** astrocytic and **(I)** microglial base processes. Data on graphs 4E/F are expressed as the Median with IQR and visualized using box and whisker plots, while data on other graphs are expressed as the Mean ± SEM and presented in the form of dot plots (*n* = 13–16/group). Comparisons among groups were performed with either the one-way ANOVA followed by Dunnett’s *post-hoc* test (data with Gaussian distribution) or the Kruskal-Wallis test followed by Dunn’s *post-hoc* test (data with non-parametric distribution). *p < 0.05, ^**^p < 0.01, and ^***^p < 0.001.

Because a recently published study demonstrated that antibiotics promotes glial cells activation in adult mice (Çalışkan et al., 2022), we next aimed to evaluated whether MAA affects astroglia and microglia in 5-week-old offspring. The morphology and density of astrocytes and microglia in the CA1 hippocampal area was evaluated using GFAP and Iba1 markers, respectively (Figures 4A, B). There was a trend to reduce the density of astrocytes (by 12%; *p* = 0.07) in 3-w antibiotics mice (Figure 4C). In addition, the surface area of GFAP^+^ cells and the size of astrocyte territories were significantly reduced by 21% (*p* < 0.001) and 18% (*p* < 0.001), respectively, in 2-w antibiotics offspring compared with that of the control offspring group (Figures 4D, E). In contrast, no significant differences in the density and surface area of Iba1^+^ microglial cells were observed in antibiotic-treated and control groups of mice (Figures 4G, H). In addition, offspring also demonstrated a significant decrease in the number of astrocytic (2nd and 3rd-w antibiotics group) and microglial (3-w antibiotics group) base processes (Figures 4F, I), which is the evidence of de-ramification and glial cells activation (Schilling et al., 2004; Althammer et al., 2020).

### 3.5. Neurogenesis in the dentate gyrus

**FIGURE 5:**
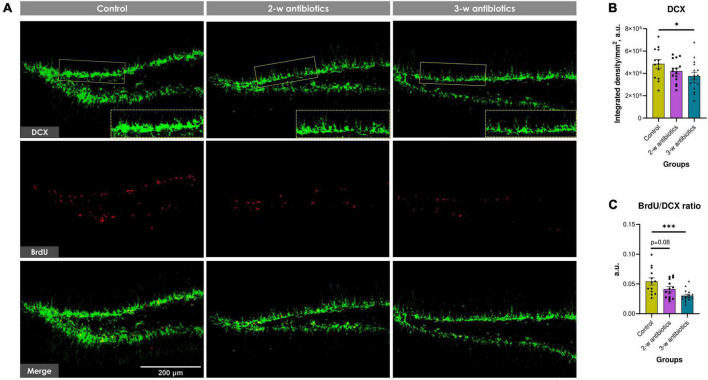
Neurogenesis in the dentate gyrus of C57BL/6J mice born to antibiotic-treated dams. **(A)** Immunofluorescent-labeled images of DCX^+^ neuroblasts (green) and BrdU^+^ proliferating cells (red) at 20× objective magnification. We used crops of DCX^+^ cells denoted by the yellow dotted line for morphometric analysis. **(B)** The integrated density of DCX^+^ pixels per mm^2^ of the subgranular zone and *stratum granulosum*. **(C)** The relative proliferation rate of neuroblasts (BrdU/DCX ratio). Data are expressed as the Mean ± SEM (*n* = 13–16/group). Comparisons among groups were performed with the one-way ANOVA followed by Dunnett’s *post-hoc* test. *p < 0.05 and ^***^p < 0.001.

To further understand the impact of MAA on exacerbated neuropathological changes observed in offspring, the level of hippocampal neurogenesis was estimated by counting DCX^+^ and BrdU^+^ pixels in micrographs of the dentate gyrus (Figure 5A). The integrated density of DCX^+^ pixels in the control group was 4.86 ± 0.39 × 10^6^ a.u. per mm^2^ of the subgranular zone and *stratum granulosum*. By contrast, the DCX^+^ density was significantly diminished by 13% and 23% (*p* < 0.05) in 2-w and 3-w antibiotics offspring, respectively, compared to control, indicating a decline in the number of neuroblasts and neurogenic potential (Figure 5B). Furthermore, the BrdU/DCX ratio in the 2-w antibiotics and 3-w antibiotics groups decreased by 24% (*p* = 0.08) and 44% (*p* < 0.001), respectively, compared to their control group (Figure 5C). These data indicated that the MAA is associated with diminished number of proliferatively active neuronal progenitors in offspring.

### 3.6. Structural synaptic plasticity

**FIGURE 6:**
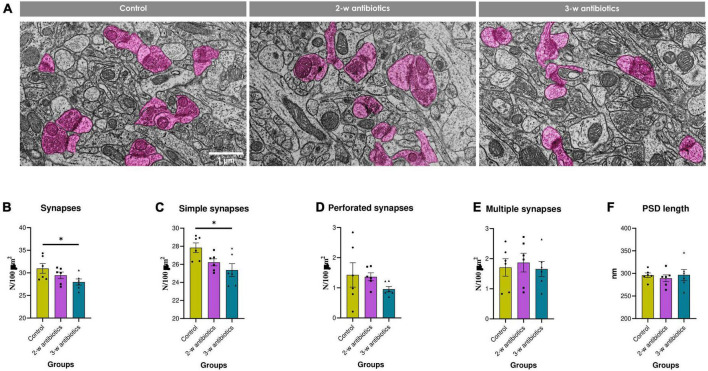
Structural synaptic plasticity in the hippocampal CA1 area of young offspring mice. **(A)** Electron micrographs of the *stratum radiatum* (×10,000). Synapses are marked in pink. **(B–F)** Morphometric characteristics of the neuropil: the number density of **(B)** synaptic contacts and their different morphological types—**(C)** simple, **(D)** perforated, and **(E)** multiple ones per 100 μm^2^ of brain tissue, as well as the **(F)** postsynaptic density length. Data are expressed as the Mean ± SEM (*n* = 6/group). Comparisons among groups were performed with the one-way ANOVA followed by Dunnett’s *post-hoc* test. *p < 0.05.

It is true that neurocognitive and emotional development is associated with synaptic structural and functional plasticity (Han et al., 2022). Therefore, we also evaluated the effects of MAA on the structural synaptic plasticity in offspring. All experimental groups were characterized by normal ultrastructure of the hippocampal neuropil, *i.e.*, synaptic contacts with clearly defined vesicles and postsynaptic density, as well as intact or moderately condensed mitochondria (Figure 6A). However, the total number of synapses and density of simple synapses in 3-w antibiotics offspring was reduced by 10% (*p* < 0.05) and 9% (*p* < 0.05), respectively, compared to control group (Figures 6B, C). No significant fluctuations in the density of perforated, multiple synaptic types, and postsynaptic density length between groups were observed (Figures 6D–F).

### 3.7. Myelination in the corpus callosum

**FIGURE 7:**
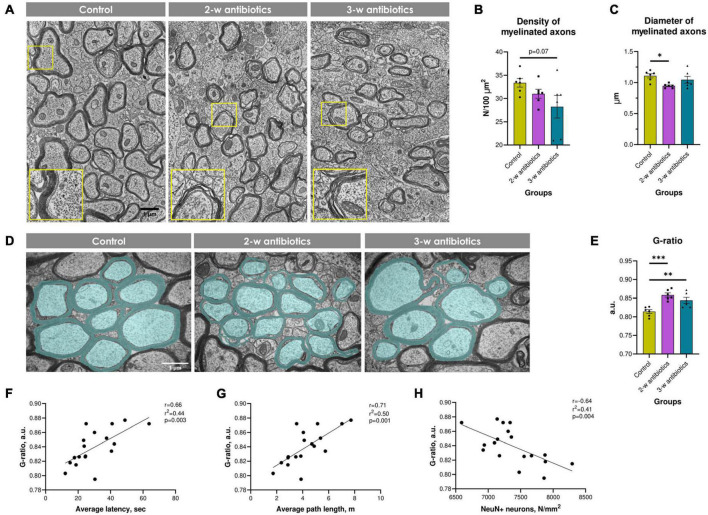
Myelination in the corpus callosum of experimental animals. **(A)** Electron micrographs of myelinated and unmyelinated axons in the corpus callosum (×4,800). Enlarged crops framed in yellow provide a more detailed morphology of myelin sheaths. **(B)** The density of myelinated axons per 100 μm^2^ of brain tissue. **(C)** Mean diameter of myelinated axons. **(D)** Electron micrographs of the corpus callosum pseudocolorized in MyelTracer software for quantification of the G-ratio: light green—axons, dark green—myelin (×6,400). **(E)** G-ratio. **(F–H)** Correlational analysis between G-ratio and **(F)** average latency during the acquisition task in MWM, **(G)** average path length in MWM, **(H)** the density of NeuN^+^ neurons in the hippocampus. Data are expressed as the Mean ± SEM (*n* = 6/group). Comparisons among groups were performed with the one-way ANOVA followed by Dunnett’s *post-hoc* test. *p < 0.05, ^**^p < 0.01, and ^***^p < 0.001.

Since our group and others demonstrated that intensive demyelination plays a critical role in the brain pathology (Pivneva et al., 2003; Chang et al., 2016; Yatsenko et al., 2020), we tested next myelination process in the corpus callosum. Morphological analysis revealed an altered myelination process in offspring exposed to antibiotics either at the 2nd or the 3rd week of gestation. This was structurally displayed in a stratification, curving, and vacuolization of myelin sheaths compared to control animals (Figure 7A). Furthermore, the density of myelinated axons decreased by 16% in 3-w antibiotics offspring (*p* = 0.07; Figure 7B), while 2-w antibiotics mice had 14% less diameter of axons (*p* < 0.05; Figure 7C) vs. control group of offspring. In addition, we detected statistically significant differences in the G-ratio values between the control (0.814 ± 0.005 a.u.) and 2-w antibiotics (0.856 ± 0.006 a.u.; *p* < 0.001) as well as 3-w antibiotics (0.844 ± 0.008 a.u.; *p* < 0.01) groups (Figures 7D, E). Therefore, these data indicate insufficient axonal myelination (hypo-myelination) in offspring under exposure to antibiotics cocktail at different time points of embryogenesis.

Finally, we tested whether hypo-myelination correlates with behavioral alterations and number of NeuN^+^ cells in offspring. To our knowledge, we detected significant positive correlations between the G-ratio and MWM average latency (*r* = 0.66; Figure 7F) and path length (*r* = 0.71; Figure 7G). In contrast, G-ratio negatively correlates with the density of NeuN^+^ pyramidal neurons in the hippocampus (*r* = −0.64; Figure 7H). Altogether, MAA affects the density and size of myelinated axons and the thickness of the myelin sheath, which correlates with behavioral and neuropathological alterations in offspring.

## 4. Discussion

Although pregnancy is considered a physiological state, most pregnant women frequently receive antibiotics to prevent maternal or neonatal complications (Siriwachirachai et al., 2014). Published clinical studies demonstrated the beneficial effects of MAA in the context of preterm labor, intrapartum fever, prevention of neonatal Group B *Streptococcus* fever, and cesarean section (de Tejada, 2014). In contrast, emerging evidence demonstrated that MAA unbalances women’s vaginal microbiota, associated with establishing the newborn gut microbiota (Zhou et al., 2020). In addition, MAA influences neuro-immune response in children and teens, leading to neurobehavioral disorders, *e.g.*, altered recognition memory responses, early types of dementia, and autism (de Tejada, 2014; Kuperman and Koren, 2016; Makinson et al., 2017; Helaly et al., 2019; Cao et al., 2021; Hickey et al., 2021; Kim et al., 2022; Madany et al., 2022a). Furthermore, antibiotic abuse is a common health hazard in Low and Middle-income countries, including Ukraine (Helaly et al., 2019; Higgins et al., 2020; Jeżak and Kozajda, 2022). A study reported that leaky gut and endotoxemia exacerbate neuroinflammation in adulthood (Alhasson et al., 2017). In addition, concerns were reported regarding the safety of antibiotics in pregnancy (Taylor-Cousar et al., 2021). Surprisingly, a study reported that delayed azithromycin treatment of mice with spinal cord injury improves their recovery (Kopper et al., 2019), indicating that antibiotics may influence neuronal dysfunction and healing. It was also reported that maternal gut dysbiosis and subsequent imbalance of bacterial metabolites correlates with cognition and neurodevelopment observed in offspring (Fröhlich et al., 2016; Madany et al., 2022b). In addition, published studies demonstrated that amoxicillin and azithromycin cross the placental barrier of mammals within two hours after their oral administration (Sutton et al., 2015; Atli et al., 2016; Zareba-Szczudlik et al., 2017; Louchet et al., 2020) and have direct impact on fetal organogenesis (Lin et al., 2012; Louchet et al., 2020). Therefore, we aimed to examine whether widely used antibiotic in gynecological practice, including amoxicillin and azithromycin, affects behavior and neurodevelopment in offspring.

In the present study, we demonstrated that exposure of pregnant dams to a cocktail of antibiotics affected the spatial reference memory and hippocampus-related spatial learning in offspring mice (Figure 2). Such results corresponded to the previously published studies showing that early-life exposure to the antibiotic in mice triggered mechanical allodynia, depressive-like behavior, and damaged spatial memory performance in MWM (Wang et al., 2021). Furthermore, it was demonstrated that antibiotics influenced the impairment of recognition memory and increased anxiety and depression in adult mice (Lurie et al., 2015; Kwon H. et al., 2020). Unfortunately, the major limitation of our findings is that no sex-specific data analysis was performed. Because antibiotic treatment during pregnancy alters offspring gut microbiota in a sex-dependent manner (Madany et al., 2022a), further studies are warranted.

In addition to behavioral changes, early published studies demonstrated that antibiotics promote gut dysbiosis-mediated intestinal hyperpermeability and BBB disruption (Fröhlich et al., 2016; Boutin et al., 2018; Lai et al., 2020). In this study, we also showed that exposure to pregnant dams during the 2nd week of pregnancy dramatically elevates intestinal permeability in offspring (Supplementary Figure 3A). Furthermore, a positive trend close to the statistical significance of the permeability of BBB was observed in those offspring (Supplementary Figure 3B). These findings corresponded to the previously published reports that both amoxicillin and azithromycin penetrate the blood brain barrier in healthy individuals even without CNS inflammation (Mingrino et al., 1981; Viaggi et al., 2022), and antibiotics may contribute to the BBB breakdown in offspring (O’Connor et al., 2021). Besides the role of BBB in central nervous protection, it also engaged in behavioral disorders (Dion-Albert et al., 2022). In addition, declined physiological barriers integrity observed under the direct impact of antibiotics and/or antibiotic-induced dysbiosis at the 2nd week of gestation may be associated with the terms of morphogenesis and formation of these barriers during embryogenesis. More specifically, the parenchyma of the murine fetal brain starts less permeable to infrared-labeled IgG2b at E15.5 to E17.5, evidencing the BBB development during this time (Braniste et al., 2014), while morphogenesis of the intestinal epithelium begins at E14.5 in mice (also the 3rd week of embryogenesis) (Kwon O. et al., 2020). Importantly, pathogen-free gut microbiota promotes healthy barriers establishment (Braniste et al., 2014). We assume that the 2nd week of pregnancy in mice might be a critical window where microbiota-shifting factors, including antibiotics, can impact some cellular or molecular targets related to the subsequent formation of both the intestinal barrier and BBB in a fetus.

As supported by the current paradigm of behavioral biology and neuroinflammation, intestinal flora dysbiosis mediated by antibiotics may influence cerebral metabolites and cause morphological and functional rearrangements of neurons and glia in brain areas controlling behavior (Guida et al., 2018). Here, we observed that the density of CA1 pyramidal neurons was diminished in offspring born to dams exposed to antibiotics during the 2nd and 3rd week of pregnancy. Neuronal loss in the hippocampus corresponded with the deterioration of spatial reference memory found in the MWM test (Figure 3). Similar to our results, it was previously demonstrated the correlation between the reduction of hippocampal neuronal density and spatial memory loss in rats (Hagberg et al., 2012; Gomez et al., 2019). Lee et al. also reported a reduced BDNF^+^/NeuN^+^ cell population in the hippocampus of antibiotic-treated mice with global forebrain ischemia (Lee et al., 2020).

A large epidemiological study demonstrated the association between MAA and an increased risk of developing various CNS disorders in the offspring via dysregulation of microglia function and myelination (Kenyon et al., 2008; Dash et al., 2022). In this study, no notable changes were observed in microglial morphology that could be linked with neuroinflammation in offspring mice; however, cellular hypotrophy of the hippocampal astrocytes and reduction in astrocyte territories were detected in young animals born from 2nd-week antibiotic-treated dams (Figure 4) that may point to compromised homeostasis in the brain tissue (Verkhratsky et al., 2019).

Meanwhile, offspring from the 3-w antibiotics group were characterized by depletion of neurogenesis in the dentate gyrus (Figure 5) and synaptic loss in the hippocampal CA1 neuropil (Figure 6). It is commonly accepted that brain synaptogenesis in mice is the most prominent from the 3rd week of embryogenesis to the 2nd week after birth (Chen et al., 2017). In addition, the 3rd week of gestation (specifically, E14.5) is dentate precursors start to migrate from the ventricular zone, and the dentate gyrus becomes distinguishable (UrbÃi̇n and Guillemot, 2014). It coincided with the most vulnerable time point to MAA we demonstrated for the postnatal hippocampal neurogenesis. Additionally, our data complement previous findings indicating a statistically significant decrease in DCX- and BrdU-labeled cells in the dentate gyrus of 4-week-old male germ-free mice (Scott et al., 2020).

Although myelination in the murine corpus callosum occurs postnatally between P14 and P45 (Sturrock, 1980), the production of oligodendrocyte precursor cells in the fetal brain begins on E12.5. It encompasses several waves till early post-embryogenesis (Chen et al., 2017). It could be partially correlated with our current observations where MAA at both the 2nd and 3rd weeks of pregnancy-induced the impairments of myelination in the corpus callosum of offspring mice (Figure 7). Furthermore, published observations suggested that either antibiotics consumption or gut microbiota depletion affects the normal myelination process in the brain. For instance, oral administration of antibiotics was shown to enhance cuprizone-induced demyelination in C57BL/6J mice (Chen et al., 2019). Lu et al. reported that myelin quantity was significantly less in the corpus callosum of 4-week-old germ-free offspring than in specific-pathogen-free mice (Lu et al., 2018).

In sum, we discovered that MAA at different times of pregnancy could pathologically alter cognitive behavior and brain development in offspring at an early age after weaning. Thus, the results of this study may lay the groundwork for developing novel therapeutic regimens for pregnant women which will protect their offspring from the development of neuroinflammation and neurodegeneration in adulthood.

## Data availability statement

The original contributions presented in the study are included in the article/Supplementary material, further inquiries can be directed to the corresponding authors.

## Ethics statement

The animal study was reviewed and approved by the Biomedical ethics committee at the Bogomoletz Institute of Physiology, NAS of Ukraine.

## Author contributions

GS, AM, and DS designed the study. DS, IO, TK, and AC performed procedures with experimental animals, behavioral tests, and sample collection. DS, IO, TK, KS, CY, SG, and GO conducted a morphological analysis of the brain. DS, AM, and GS interpreted the results. All authors contributed to the writing and revising of the manuscript.
