# Supplementary material for: Maternal antibiotic administration during gestation can affect the memory and brain structure in mouse offspring
Source: Front Cell Neurosci. 2023 May 10;17:1176676. doi: 10.3389/fncel.2023.1176676 (PMC10206017; doi:10.3389/fncel.2023.1176676)
Supplement: Supplementary file 1 [file Data_Sheet_1.pdf]

Supplementary Material

Maternal antibiotic administration during gestation can affect the memory and brain structure in mouse offspring

Dmytro Shepilov*, Iryna Osadchenko, Tetiana Kovalenko, Chiaki Yamada, Anastasiia Chereshynska, Kateryna Smozhanyk, Galyna Ostrovska, Stanislav Groppa, Alexandru Movila*and Galyna Skibo

*** Correspondence:** Dmytro Shepilov: shepilov@biph.kiev.ua; Alexandru Movila amovila@iu.edu

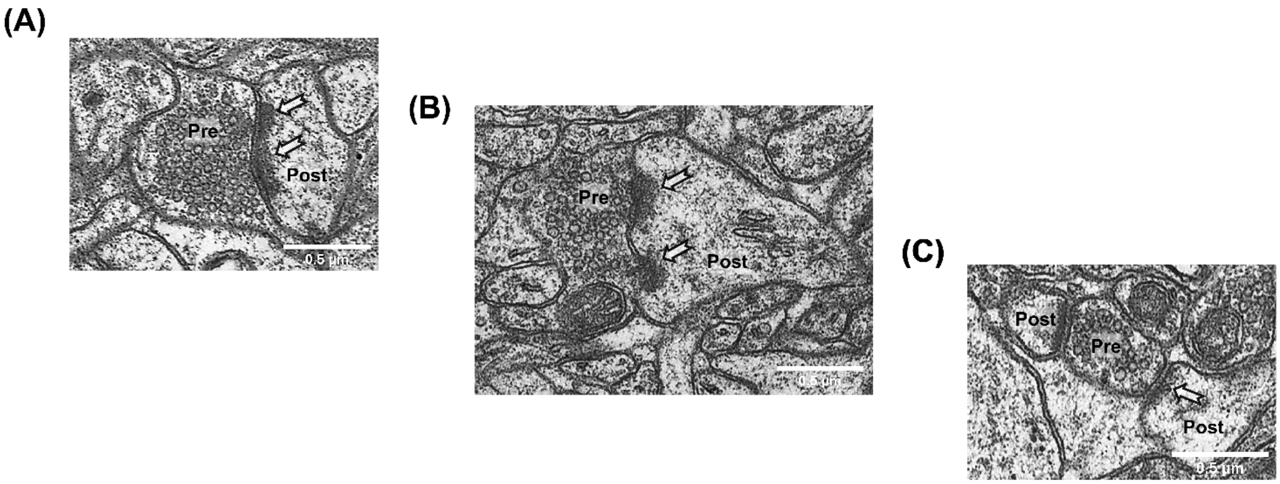

Supplementary Figure 1. Representative images of (A) simple, (B) perforated, and (C) multiple synaptic types. Pre – presynaptic terminal, Post – postsynaptic terminal. Arrows point to the postsynaptic density.

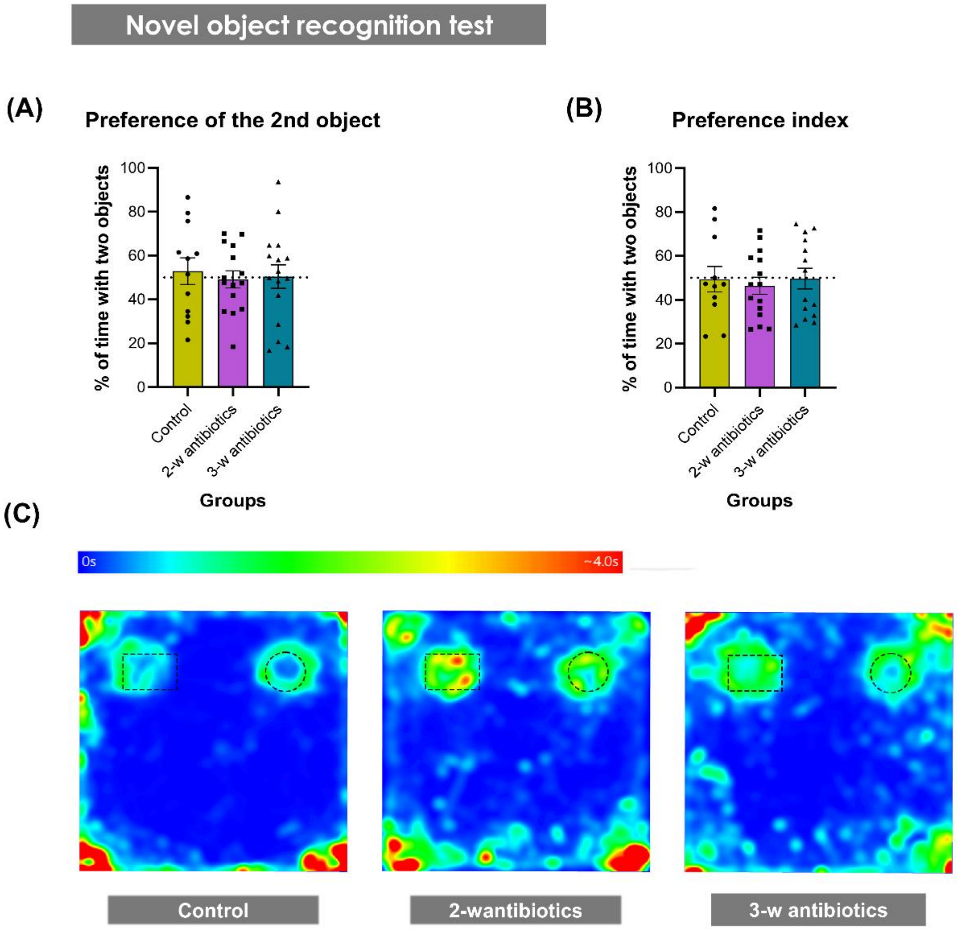

Supplementary Figure 2. Impact of maternal antibiotic administration (MAA) on associative memory in offspring. The control ***group represents offspring born from dams exposed to*** sterile drinking water over the entire period of gestation. The ***2-week antibiotics offspring are mice born from dams*** exposed to an antibiotic pharmaceutic grade cocktail containing a mixture of amoxicillin (205 mg/kg bw/day) and azithromycin (51 mg/kg bw/day) in drinking water starting from the second week of pregnancy (E8-E14); pregnant dams were treated by the antibiotic cocktail for 7 days followed by sterile (no antibiotic containing) drinking water until birth. Mice from the ***3-w antibiotics*** received antibiotics cocktail in drinking water from the 3rd week of pregnancy to delivery. (A-C) Novel object recognition test: (A) preference of the 2nd object during familiarization, (B) preference index, and (C) heat maps of animals' trajectories during the test task. The dotted line rectangle on the heat map corresponds with a familiar object, while the dotted line circle indicates the location of a novel object. Data are expressed as the Mean ± SEM and presented as dot plots (n=13-16/group). Comparisons among groups were performed with the one-way ANOVA followed by Dunnett's post hoc test.

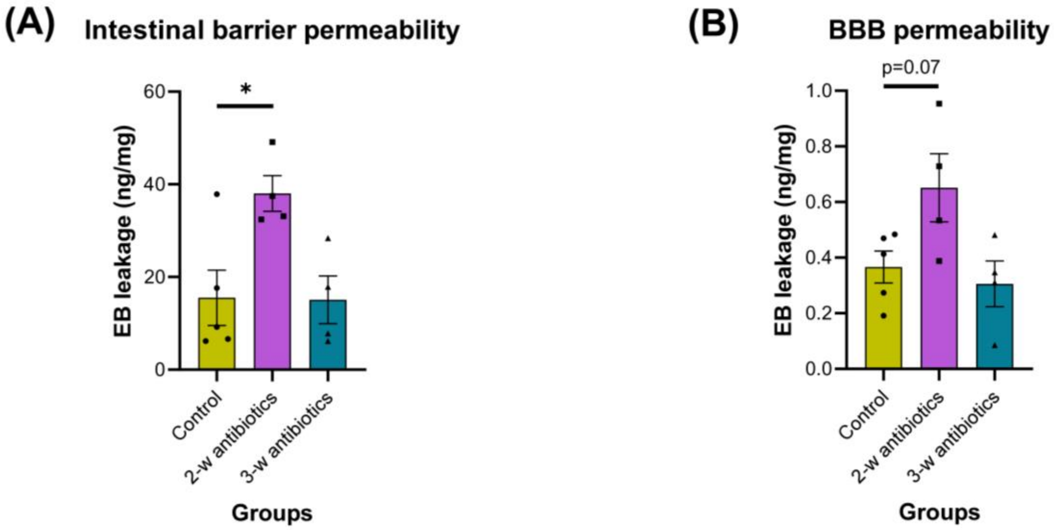

Supplementary Figure 3. Permeability of the intestinal barrier and blood-brain barrier (BBB) in offspring born from dams exposed to an antibiotic cocktail in drinking water either at the 2nd or 3rd week of pregnancy. The control group of dams was treated with sterile water (See Suppl. Fig 2 for details). The levels of Evans Blue leakage in the (A) intestinal and (B) brain tissues are presented in the form of dot plots. Data are expressed as the Mean ± SEM (n=4-5/group). Comparisons among groups were performed with the one-way ANOVA followed by Dunnett's post hoc test. *$p<0.05$ indicates significant differences vs. control.
